# Predicting attention shifting abilities from self-reported media multitasking

**DOI:** 10.3758/s13423-018-01566-6

**Published:** 2019-04-27

**Authors:** Pia Elbe, Daniel Eriksson Sörman, Elin Mellqvist, Julia Brändström, Jessica K. Ljungberg

**Affiliations:** Department of Psychology, Umeå University, SE- 90187 Umeå, Sweden

**Keywords:** Media, Multitasking, Switching, Executive functions, Attention

## Abstract

Media multitasking is an increasingly prominent behavior in affluent societies. However, it still needs to be established if simultaneous use of several modes of media content has an influence on higher cognitive functions, such as divided attention. In this study, attention shifting was the primary focus, since switching between tasks is assumed to be necessary for media multitasking. Two tasks, the number–letter and local–global task, were used as measures of switching ability. The cognitive reflections task was included to control for possible effects of intelligence. Results from linear regression analyses showed that higher levels of media multitasking was related to lower switching costs in the two attention-shifting tasks. These findings replicate previous findings suggesting that heavy media multitaskers perform better on select measures of task switching. We suggest two possible explanations for our results: media multitasking may practice skills needed for switching between tasks, or high media multitaskers are choosing this style of technology use due to a dominating personality trait in this group.

There is no consensus about the effects of media multitasking, or the simultaneous use of several streams of media, on higher cognitive functions. But with the opportunities for using multiple forms of media increasing, finding out if regularly consuming several modes of electronic content impacts various higher cognitive functions is a pressing task.

One questionnaire, the Media Multitasking Index (MMI; Ophir, Nass, & Wagner, 2009), dominates media multitasking research and acts as an acceptable measurement tool for media multitasking frequency and behavior. It is a self-report survey that asks respondents to report how many types of media they simultaneously consumes, and was originally developed to compare levels of media multitasking with several tests of cognitive control. In that study, two indicators of executive functioning were used. First, a switching task (number–letter classification) showed that light media multitaskers had lower task-switching costs (i.e., difference in response times between switch trials and nonswitch trials) compared with heavy media multitaskers. Second, high media multitaskers showed a greater decrease in performance comparing performance from a two-back to a three-back updating task. The authors suggested that differences in performance could be because high media multitaskers are less capable of filtering out irrelevant representations in memory, similar to the mechanism of filtering out irrelevant stimuli in attentional control. What should be noted is that the MMI has been modified and updated in later studies because of continuous changes in how people interact with technology in their daily life.

There have been several studies which have attempted to replicate Ophir et al.’s (2009) original findings of a relationship between the MMI and cognitive functioning, with varying results. There is a definite lack of consensus about the impacts of frequent media multitasking, so much so that expert clinicians tend to warn against multitasking and suggest doing tasks consecutively (for a review, see Uncapher et al., 2017). Finding the extent of cognitive differences between high and low media multitaskers therefore has the potential to inform policy.

Before motivating the design of the present study, previous research linking media multitasking and attention should be explored. It should be stressed that studies investigating specific executive functions and the possible impacts of media multitasking exhibit disparate findings that are difficult to compare directly. Some studies have confirmed a negative link between media multitasking and attentional control, particularly in self-reported assessments of attentional control (e.g., Baumgartner, Weeda, van der Heijden, & Huizinga, 2014). Behavioral assessments in the lab of attention *inhibition* have mostly confirmed the negative impacts of high media multitasking (Cain & Mitroff, 2011; Minear, Brasher, McCurdy, Lewis, & Younggren, 2013). Attention inhibition is an aspect of attention where intrusive or irrelevant stimuli need to be avoided. In contrast, attention *switching*, which is also part of the attentional control network, has mixed results in regards to media multitasking. Attention shifting, measured using task-switching behavioral tests in the lab, refers to an aspect of executive functions where individuals must refocus their attention quickly between stimuli or tasks.

Several studies suggest that frequent media multitasking is harmful to attention shifting. A recent study on media multitasking and task switching used the same cognitive task as the original study by Ophir et al. (2009) found higher switching costs while performing the number–letter task in the high media multitasking group compared with the low media multitasking group (Wiradhany & Nieuwenstein, 2017), and thus replicated the findings from Ophir et al. (2009). Ralph, Thomson, Cheyne, and Smilek (2014) asked participants to rate their own attentional control and media multitasking frequency. They asked their participants to complete subjective measures of their own lapses in attention and cognitive control. A number of validated scales—that is, the Mindful Attention Awareness Scale–Lapses Only (MAAS-LO); Attention-Related Cognitive Errors Scale (ARCES); Memory Failures Scale (MFS); spontaneous and deliberate mind wandering; attentional control: switching (AC-S), distractibility (AC-D); and Media Multitasking Beliefs Questionnaire (MMBQ)—were used. High media multitaskers rated themselves more poorly on all these measures. This finding with self-reported data, coupled with behavioral data from the Ophir et al. (2009) and Wiradhany and Nieuwenstein (2017), suggests that high levels of media multitasking may have negative impact on select attentional resources.

It should be noted that another study has also examined the impact of media multitasking on task switching and found a strong disadvantage of frequent media multitasking (Gorman & Green, 2016). However, although a relationship between task switching and media multitasking was a main finding, the aim of the study was to see if there was an advantage of a controlled-breathing mindfulness exercise, performed between task-switching trials. Interestingly, this short mindfulness training was effective for improving the speed of high media multitaskers in the task-switching trials but not beneficial for low media multitaskers. The impact of the mindfulness training shows that short-term behavioral change can impact attention shifting in relation to media multitasking.

Positive effects of media multitasking on attention switching have been reported (for a review, see van der Schuur, Baumgartner, Sumter, & Valkenburg, 2015). Alzahabi and Becker (2013), for instance, instead found high media multitaskers to be significantly better at the number–letter task, similar to the task that Ophir et al. (2009) employed. In addition, they included a measure of dual-task ability, in which participants had to perform both elements (i.e., in a trial where “both” is indicated, participants respond for number and letter) of the switching task at the same time. The authors found no difference between groups in this task. Therefore, the authors concluded that high media multitaskers may be better at switching between tasks, but not better at maintaining attention on two tasks at once.

In a study by Cardoso-Leite et al. (2016) on the effects of technology consumption on different aspects of cognition, including task switching, the authors found improved task-switching performance for media multitaskers among individuals who do not play video games, which supports the positive findings of Alzahabi and Becker (2013) for high media multitaskers. However, in the action-video-game-playing group, low media multitaskers performed better on task switching, supporting Ophir et al. (2009). Although the authors in the Cardoso-Leite et al. study (2016) caution that their findings are not conclusive due to small sample sizes (see also a meta-analysis by Wiradhany & Nieuwenstein, 2017, for similar conclusions), they suggest that a nuanced approach will be needed to fully tease apart the variation in media multitasking and attention-shifting performance for special groups, such as action video gamers.

It has been suggested that tasks requiring skills similar to media multitasking may be the ones exhibiting the greatest benefit. For example, Lui and Wong (2012) found that the pip-and-pop task for multisensory integration, where spatial auditory events guide visual attention, correlated positively with the MMI, and concluded that certain types of multisensory functions may benefit from media multitasking. This positive correlation points to the idea that individuals with high scores on the MMI are more efficient at refocusing their attention between tasks. These findings support the idea that there may be a training effect of media multitasking on attention shifting, but no benefit for attention inhibition, which requires periods of focused attention, unlike media multitasking behavior.

To help unravel which factors of attention switching affect media multitasking, Alzahabi, Becker, and Hambrick (2017) performed factor analyses on several task-switching paradigms. They found an advanced preparation factor, which refers to the participant’s ability to prepare for the trial type after receiving a cue, and a passive decay factor, which refers to attention on the previous task carrying over into the current task. A main finding was that frequent media multitasking was related to advanced preparation.

Recently, Schutten, Stokes, and Arnell (2017) suggested that higher levels of media multitasking are related to greater impulsivity and less self-control, showing that heavy media multitaskers had a decision-making style that was more intuitive, compared with light media multitaskers, who had a decision-making style that was more analytic and thus also more effortful, as indicated by performance in the cognitive reflection test (CRT; Frederick, 2005). These separate ways of reflecting around problems is sometimes referred to as System 1 and System 2 thinking (Kahneman, 2011), where the former is supposed to reflect fast and intuitive thinking, and the latter slower, deliberate, and more effortful reflecting thinking. The CRT is also correlated to common measures of intelligence—for instance, the Raven’s Standard Progressive Matrices and Set I of Raven’s Advanced Progressive Matrices (Primi, Morsanyi, Chiesi, Donati, & Hamilton, 2016), and individual differences in executive functioning have, in turn, been linked to individual differences in intelligence (e.g., Duggan & Garcia-Barrera, 2015; Duncan, Emslie, & Williams, 1996; Friedman et al., 2006). The inclusion of the CRT is therefore warranted in studies aimed to investigate the relationship between media multitasking and attention shifting.

Given the mixed findings from previous studies, the overall aim of this study was to investigate the relationship between media multitasking and the dimension of higher cognitive functioning concerned with attention switching. The number–letter and local–global task were used as indicators of task-switching ability, and the MMI (Ophir et al., 2009) was used as measure of media multitasking. In addition, the potential influence of thinking/intelligence were considered using the CRT. The present study addressed the following questions: Can level of media multitasking predict task-switching performance? Can thinking/intelligence predict task-switching performance? Is there an interaction effect between media multitasking behavior and thinking/intelligence, predicting task-switching performance? Is thinking/intelligence related to levels of media multitasking?

## Method

### Participants

Fifty-one individuals (20 men and 31 women) participated in this study, resulting in an age range of 17 to 35 years, and with a mean age of 24.1 years (*SD* = 3.3) years. All participants signed an informed consent and were reimbursed for their participation. This research was approved by the Regional Ethical Committee, Umeå, Sweden.

### Materials

#### Media Multitasking Index

A revised version of the MMI) Ophir et al., 2009) was chosen as a measure for frequency of media multitasking. This included 11 different forms of media tasks, instead of the original 12. “Mobile texting” was deleted because of its similarity to “instant messaging.” First, for each activity (e.g., text messaging, e-mail, video games, Web surfing), the respondents first replied to how many hours per week they used these different forms of media. Second, for each type of (primary) media, they reported how often they used other media while participating in the primary media. Ratings were performed on a 4-level ordinal scale including the alternatives *never, sometimes, often,* and *almost always*. To calculate scores, each of the 144 items “How often do you use [media] with [media]?” was summed in each category. Then this sum was multiplied by the number of hours spent each week using the media type. The result was then divided by the total number of hours spent consuming all of the media types per week. This calculation was repeated for all 11 media types, and the resulting scores were added up to yield the MMI score used in our analyses. This formula is identical to the one used in the original Ophir et al. (2009) study, except for the absence of “mobile texting” as a category:

1 $$ MMI={\sum}_{i=1}^{12}\frac{m_i\times {h}_i}{h_{total}}, $$

The MMI was analyzed as a continuous variable. Studies involving the MMI have divided their participants into high and low media multitaskers in the past by taking a percentage or fraction of multitaskers from the top and bottom of the range of scores. Since analyzing these extremes does not account for variation in the normal range of medium multitaskers, this study analyzes all participants’ MMI scores.

#### Attention-shifting tasks

**Fig. 1:**
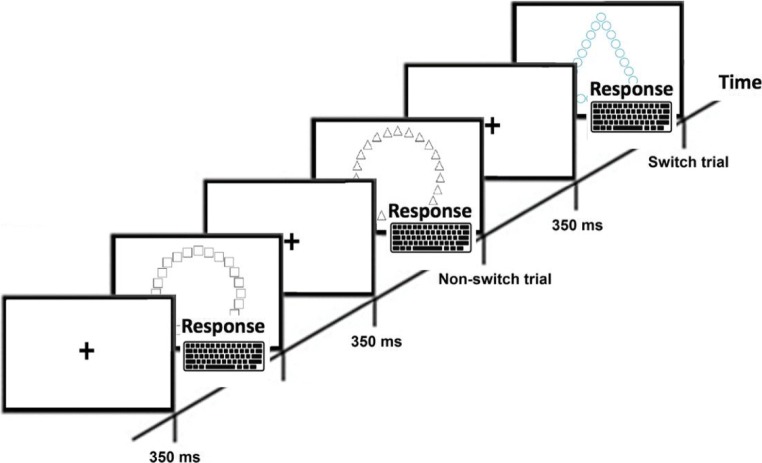
Illustration of the local–global task. If the stimulus was in black color, the task was to decide the shape of the global figure. If the stimulus was blue, the task was to make a decision about the local figure(s). Attention switching occurred when shifting between local and global

**Fig. 2:**
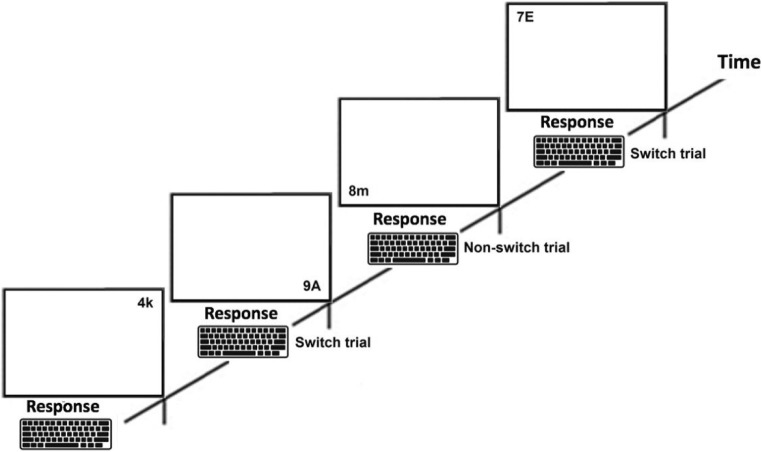
Illustration of the number–letter task. If a number–letter combination was presented in any of the upper corners of the screen, the participant should decide if the number as odd or even. If the combination displayed in any the bottom corners, the task was to decide if the letter was lowercase or capital. Attention switching occurred when between categorizing letters and numbers

In order to measure attention shifting, two tests were used: the number–letter task and the local–global task. For task illustrations, see Figs. 1 and 2. Both tasks require participants to switch focus depending on a certain aspect of their appearance (Miyake et al., 2000; Rogers & Monsell, 1995). In the number–letter task (Rogers & Monsell, 1995), participants were presented with a number and a letter. The height of the stimuli was approximately 0.8 inches. Depending on the spatial location of the stimulus on the screen, individuals were required to respond by pressing buttons on a keyboard to either categorize the number (odd or even) or the letter (capital or lowercase). If a number–letter combination appeared in the top left or top right quadrant of the monitor, the respondent should categorize the number, and if displayed in the bottom left or bottom right quadrant, the respondent should categorize the letter. In both conditions, the *X* and *M* keys on a standard keyboard were used. In the first block (32 trials), the number–letter combination was located only in the top quadrants of the monitor, and thus participants focused only on the numbers. Participants pressed the *X* key for odd numbers, and the *M* key for even. In the second block (32 trials), stimuli were displayed only on the bottom quadrants of the screen, and hence participants focused only on the letters. Participants were instructed to press the *X* key for lowercase letters, and the *M* key for capital letter. In the final mixed block (128 trials), randomized number–letter combination appeared in all quadrants of the screen, and thus, for some trials attention shift was required between classification of numbers and letters. The number of switch and nonswitch trials was the same .

**Fig. 3:**
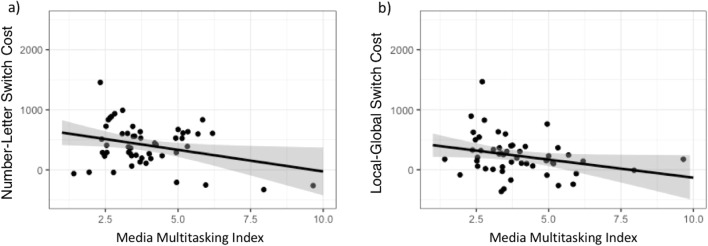
Scatterplot illustrating the relationship between (**a**) Media Multitasking Index (MMI) score and number–letter switch costs, and (**b**) MMI score and local–global switch costs. Black line is a linear regression line. Gray shaded region surrounding it denotes the 95% confidence area

The local–global task (Navon, 1977) similar to the one used by Miyake et al. (2000) was used as second measure of attention shifting. In each trial, a fixation cross was first shown for 350 ms in the center of the screen and then replaced by a figure that either was a triangle, a square, a circle, or a cross. The height of this “global” figure was approx. 6.3 inches. This figure was in turn composed of many smaller “local figures. The “local” shape could be consistent or inconsistent with the “global” shape. If the stimulus appeared in black color on the screen, the respondent was instructed to make a decision about the global shape, and if the stimulus appeared in blue on the screen, the respondent was instructed to make a decision about local shape. Thus, if the color of the stimulus switched, attention switch was required from local to global, and vice versa. In nonswitch trials participants performed the same categorization as in previous stimuli (i.e., global to global, or local to local). The participants were instructed to press the “1” key (i.e., 1 line) if the correct response was a circle, the “2” key (2 lines) for a cross, “3” key (3 lines) for a triangle, and “4” key (4 lines) for a square. After 38 practice trials, participants performed 98 test trials in prerandomized order. The number of switch and nonswitch trials was equal throughout the experiment, and each trial was separated by a 500-ms response-to-stimulus interval. None of the participants reported vision impairments, so correct color identification can be assumed. Both tasks were performed on 24-inch monitors.

For both tasks, switch cost was calculated as the difference in response times between correct responses on switch trials (change in categorization/focus compared to previous trial) and correct responses on nonswitch trials (same categorization/focus as in previous trail). A larger positive difference in reaction times indicates that more effort was needed when shifting was required compared with the straightforward nonshifting trials. This difference will be known as mean switch cost.

#### Cognitive reflections test

The CRT, used as measure of thinking/intelligence, was administered in six different versions with randomized order. The test contains six word problems that warrant numeric answers that can best be achieved by using analytic reasoning instead of one’s first intuition (Frederick, 2005; Primi et al., 2016). Each question was presented on a separate page, and participants were not allowed to turn back to any previously answered question. The maximum time limit to solve all questions was 9 minutes. The number of corrects answers was used as dependent variable in the analyses. The CRT correlates positively with common measures for intelligence, namely, Raven’s Standard Progressive Matrices and Set I of Raven’s Advanced Progressive Matrices, among others (Primi et al., 2016), which validates the use of the CRT as a useful short-form measure.

### Procedure

Before starting the experiment, the participants were given both oral and written instructions about the tasks and the procedure. All participants were allowed to conduct test trials before each task session to be able to get familiarized with the tasks. They were also given a written memorabilia as a reminder of which button to press if they forgot this during the test. After finishing the cognitive tasks, the participants performed the CRT. Estimated time to complete all three tests was approximately 45 minutes.

## Results

Descriptive statistics based on participant performance are presented in Table 1.

**Table 1:** Means, standard deviations and min/max values for all variables used

	*N*	Minimum	Maximum	Mean	Median	*SD*
Age (years)	51	17.00	35.00	24.08	24.00	3.35
Female (%)	51	–	–	60.8	–	–
Media Multitasking Index	51	1.41	9.65	3.91	3.51	1.51
Local–global switch cost (ms)	51	−361.61	1,468.45	234.75	195.08	327.79
Number–letter switch cost (ms)	51	−328.64	1,457.42	412.01	409.79	352.61
Cognitive reflections test (Acc)	51	0.00	6.00	2.63	2.00	1.95

ms = milliseconds; Acc = accuracy

Next, zero-order correlations were performed between MMI, switch costs in the local–global and the number–letter tasks, performance in the CRT, gender, and age. Correlations between variables are presented in Table 2.

**Table 2:** Zero-order correlations between scores on the MMI, age, gender, number–letter switch cost, local–global switch cost, and CRT

*n* = 51	Age	Gender	MMI	Local–Global	Number–Letter	CRT
Age	–	−0.01	0.22	0.04	0.06	−0.03
Gender		–	−0.05	0.06	−0.06	−0.22
MMI			–	−0.28*	−0.31*	−0.21
Local–global switch cost				–	0.21	−0.19
Number–letter switch cost					–	−0.02
CRT						–

MMI = Media Multitasking Index; CRT = cognitive reflection test; * *p* < .05

Results showed a significant negative correlation between MMI and switching costs in the number–letter task (*p* < .05) and a significant negative correlation between MMI and switching costs in the local–global task (*p* < .05), revealing that individuals who rated higher in levels of media multitasking performed better (i.e., had lower switch costs) in both task (Fig. 3). No relationship could be confirmed between performance in the CRT and any of the other variables. Participants’ age and gender did not affect any of the measured variables.

Finally, as all interval level data passed the Shapiro–Wilks test for normality, as well as a visual test, a linear regression analysis was performed. Two linear regression models were used, with scores on the MMI as the predictor. The first model had MMI score as predictor for number–letter switching cost. Here, MMI was significant as predictor for number–letter task switch costs (β = −0.308), *F*(1, 49) = 5.159, *p* = .027, and naturally the adjusted *R*^2^ (0.077) was significant (*p* = .027). The second model had MMI as predictor for local–global switching cost. The second model was significant, as indicated by the adjusted *R*^2^ coefficient 0.058 (*p* = .048). MMI was significant as predictor for local–global switching cost (β = −0.277), *F*(1, 49) = 4.08, *p* = .048. Finally, after centering the predictor variables, no interaction effect was found between MMI and CRT on performance in the number–letter task.

## Discussion

In this study, we wanted to provide answers of the following questions: Can level of media multitasking predict task-switching performance? Can thinking/intelligence predict task-switching performance? Is there an interaction effect between media-multitasking behavior and intelligence predicting task-switching performance, and is thinking/intelligence related to levels of media multitasking? First, the results from this study show that higher levels of media multitasking is related to lower switching costs in the number–letter task and in the local–global task, both of which are used as measures of task-switching ability (i.e., those individuals who multitask more frequently had better scores in the cognitive tasks). Second, performance in the CRT, used as measure of thinking/intelligence, was not related to task-switching ability or to media multitasking.

Results from this study do not support the notion that participants who report more frequent practice in media multitasking also perform worse, on average, on attention-shifting tasks. Thus, these results do not support findings suggesting that frequent media multitaskers are worse at cognitive control, including attention switching (see, e.g., Loh & Kanai, 2014; Lui & Wong, 2012; Ophir et al., 2009; Ralph et al., 2014), and who posit that high media multitaskers may be worse at suppressing irrelevant tasks, and therefore do worse on task-switching behavioral tests. Rather, this study gives further evidence to a switching benefit in line with more recent studies (Alzahabi & Becker, 2013; Alzahabi et al., 2017). A significant relationship between media multitasking and both cognitive tasks helps support the conceptual significance of this result.

The findings of this study combined with the studies on inhibitory control (Baumgartner et al., 2014; Minear et al., 2013; Ophir et al., 2009), suggests that increased media multitasking is correlated with increased ability to switch attention, but the opposite is true for inhibiting attention. High media multitaskers may over time become more susceptible to distractors (Cain & Mitroff, 2011), but also increase in their ability to switch quickly between different stimuli, which is measured by the number–letter and the local–global tasks used here. This clearly makes it impossible to find one answer to the question if media multitasking enhances or diminishes attentional control over time, because there are differential impacts depending on which type of attention is measured. This has been observed by Lin (2009), who commented that high media multitaskers may have a breadth bias. This means that individuals who divide their attention between multiple tasks may be biased to a broader way of processing their environment, compared with individuals who focus their attention and perform well on cognitive control tasks in the lab (depth bias). Although we did not find negative effects on cognitive control, this theoretical understanding of breadth and depth biases may still hold. The ability to focus attention during inhibition tasks may be aided by a depth bias, whereas the ability to switch attention during shifting tasks may be aided by a breadth bias.

The finding that performance on the CRT was not independently (or in interaction with MMI) related to switching ability suggests that the effects of media multitasking on switching ability was independent of thinking style/intelligence. This can, perhaps, be perceived as surprising if only considering CRT as a proxy of intelligence. Then, the result speaks against some previous findings of the relationship between executive functioning and intelligence (see, e.g., Duggan & Garcia-Barrera, 2015; Duncan et al., 1996; Friedman et al., 2006). However, as noted, the CRT is not an exhaustive measure of intelligence. Thus, if solely interested in the impact of intelligence and critical thinking, future studies should include more sensitive instruments. In this study, we did not find an association. There is other evidence that Raven’s Progressive Matrices, another proxy of intelligence, is only associated with media multitasking and attention switching when advanced preparation in the form of a cued trial is involved (Alzahabi et al., 2017). This suggests that intelligence may play a role in the advanced preparation aspect of attention switching, but not in a noncued paradigm, such as the one we employ here.

One important difference between our study and others previously mentioned (Cardoso-Leite et al., 2016; Ophir et al., 2009; Ralph et al., 2014; Wiradhany & Nieuwenstein, 2017) is that we did not fit participants into binary groups of high and low media multitaskers. In this study, the MMI was treated as a continuous variable, although this is not the original scoring mechanism into high and low, as used by Ophir et al. (2009). The original scoring mechanism includes only results one standard deviation above and below the mean, which means excluding the middle section of scores in order to obtain two categories of media multitaskers. Our decision was made for the following reason: conceptually, the findings should be generalizable to as many media users as possible, not just to extreme cases. Also, finding a significant effect in a continuous analysis is more difficult than analyzing only the extreme cases. In studies that also used continuous measures of the MMI (Alzahabi & Becker, 2013; Alzahabi et al., 2017), the findings are similar to this study, although these researchers used an extended version of the MMI that included other activities that could be done in conjunction with media interactions, such as physical exercise.

This study also differs from other studies in how outliers were assessed (Alzahabi & Becker, 2013; Alzahabi et al., 2017; Minear et al., 2013). For instance, in this study, individuals with negative switching costs were not excluded. Also, no subjects were excluded based on very high MMI scores in this study. In contrast, Alzahabi and Becker’s (2013) motivation for excluding individuals who reported more than 165 hours per week of media use is that this would add up to almost 24 hours of media use per day. However, we believe that that it was necessary for our participants to be able to report hours of media use separately for each type of media on the index, regardless of simultaneous media use. Therefore, if an individual uses two or more media simultaneously for most of the time spent with media, this will be reflected in the total number of hours spent using media. This conceptual difference in interpretation of the MMI scores may have some impact on our results.

In this study, compared with other task-switching paradigms (Rogers & Monsell, 1995), our attentional tasks did not involve a cue for the upcoming trial type. Participants thus could not prepare for the task in advance. It has been suggested that high media multitaskers have a specific advantage for the mechanism for preparing to switch tasks quickly once a cue is given (Alzahabi et al., 2017). No such mechanism exists in our design, because no cue is given prior to each trial. Despite this, higher media multitasking was associated with lowered switch costs. Therefore, our findings indicate that there is another behavioral component in addition to advanced preparation that frequent media multitaskers benefit from.

Some studies using attention-shifting tasks have analyzed accuracy and found higher accuracy for high media multitaskers (Alzahabi & Becker, 2013; Cardoso-Leite et al., 2016); however, not all studies have been able to confirm this relationship (Minear et al., 2013). Future studies would possibly benefit by disentangling how accuracy in the switching tasks is related to media multitasking and to learn more if a relationship between media multitasking and attention switching is affected by accuracy in both switching and repeating trials. With regard to the switching tasks used, it should also be stressed that our data revealed a nonsignificant correlation (*r* = .21, *p* = .13) between the tasks. Although it is possible that a significant correlation would have been present with greater statistical power, a poor relationship between tasks supposed to tap the same executive function is not uncommon. Previous studies have found low correlations between executive tasks (see, e.g., Paap & Sawi, 2014; Bakker et al. 2012), and thus more research is needed to develop tests of cognitive control with better task validity. However, since this study still found a relationship between media multitasking and both these measures, it is plausible that these measures, at least to some extent, may reflect some aspects of the same construct.

Despite being the most common measure, using the mean difference in reaction times between switch and nonswitch trials has been questioned (Hughes, Linck, Bowles, Koeth, & Bunting, 2014). The reliability of switch-cost measures for attention-shifting tasks has been analyzed in detail by Alzahabi et al. (2017), who find their switch-cost measures to have low reliability. They opted to perform a *z*-score transformation technique to estimate switch costs. Recently, additional ways of measuring switch-cost reliability have emerged. Some suggest that because a high correlation between the switch and nonswitch elements indicates low reliability, the true correlation should be taken into consideration to help gauge the observed correlation in difference score elements (Trafimow, 2015). This observation increases the actual reliability of difference scores, but makes comparing the reliability of these measures to other measures more difficult.

Some limitations of this study should be considered. A convenience sample of relatively young individuals with a narrow age range was used (*M* = 24.1 years, *SD* = 3.35). Thus, seeing if there is any interaction of age with the MMI was not possible. This would be of interest, because of perceived generational differences in technology use (Gell, Rosenberg, Demiris, LaCroix, & Patel, 2015; Thompson, 2013). How adolescents and young adults interact with digital media seems to differ from how older adults interact with digital media, and if this is reflected in scores on the MMI, then it is not known to the authors of this study. Another limitation is that information about more specific media use was not collected. As previously mentioned, one study on executive functions and media multitasking also included action-video-game players (Cardoso-Leite et al., 2016). Future studies should aim to include more information concerning technology use, because multitasking with video games is likely a much different experience compared with multitasking with more passive forms of media consumption, such as listening to music or watching TV.

In sum, this study lends further evidence that higher levels of media multitasking are negatively correlated with processing costs in attention switching, which could suggest that individuals who tend to use several media simultaneously also tend to get better at attentional control skills, on average. This implies that there may be a practice effect of frequent media multitasking on shifting attentional control. It is necessary to repeat here that it cannot be determined here if this is a causal relationship or if there is a relationship between media multitasking frequency and a predisposition to score well in the attention-shifting tasks. Importantly, these findings also add evidence that some advantageous process besides advanced preparation from trial-type cues is present in frequent media multitaskers. Further studies should examine what these attentional control mechanism are in more detail. Future research could also include other personality factors that might influence media multitasking behavior, such as sensory processing sensitivity (Aron & Aron, 1997) or introversion–extraversion personality traits, to understand what personality factors may be responsible for the relationships found in this study (Duff et al. 2014). Discovering those personality factors could help individuals optimize their media multitasking behavior. Finally, for future research, it may also be relevant to include participants from populations with attentional deficits (Seo et al. 2015), or similar, to understand what influence the use of simultaneous media has on higher cognitive functions.
